# H5N2 Highly Pathogenic Avian Influenza Viruses from the US 2014-2015 outbreak have an unusually long pre-clinical period in turkeys

**DOI:** 10.1186/s12917-016-0890-6

**Published:** 2016-11-22

**Authors:** Erica Spackman, Mary J. Pantin-Jackwood, Darrell R. Kapczynski, David E. Swayne, David L. Suarez

**Affiliations:** Southeast Poultry Research Laboratory, USDA-Agricultural Research Service, 934 College Station Rd, Athens, GA 30605 USA

**Keywords:** Highly pathogenic avian influenza virus, Clade 2.3.4.4 H5N2, Turkey disease, Avian influenza outbreak, Chicken disease

## Abstract

**Background:**

From December 2014 through June 2015, the US experienced the most costly highly pathogenic avian influenza (HPAI) outbreak to date. Most cases in commercial poultry were caused by an H5N2 strain which was a reassortant with 5 Eurasian lineage genes, including a clade 2.3.4.4 goose/Guangdong/1996 lineage hemagglutinin, and 3 genes from North American wild waterfowl low pathogenicity avian influenza viruses. The outbreak primarily affected turkeys and table-egg layer type chickens. Three isolates were selected for characterization in turkeys: the US index isolate from December 2014 (A/northern pintail/WA/40964/2014), and two poultry isolates from April 2015 (A/chicken/IA/13388/2015 and A/turkey/MN/12528/2015).

**Results:**

Four week old broad-breasted white turkeys were inoculated with one of three doses (10^2^, 10^4^ or 10^6^ 50% egg infectious doses [EID_50_] per bird) of each of the isolates to evaluate infectious dose and pathogenesis. The mean bird infectious dose of A/northern pintail/WA/40964/2014 and A/turkey/MN/12528/2015 was 10^5^ EID_50_ per bird, but was 10^3^ EID_50_ per bird for A/chicken/IA/13388/2015, suggesting the latter had greater adaptation to gallinaceous birds. All three isolates had unusually long mean death time of 5.3–5.9 days post challenge, and the primary clinical signs were severe lethargy and neurological signs which started no more than 24 h before death (the average pre-clinical period was 4 days). Infected turkeys also shed high levels of virus by both the oropharyngeal and cloacal routes.

**Conclusions:**

The unusually long mean death times, high levels of virus in feces, and increased adaptation of the later viruses may have contributed to the rapid spread of the virus during the peak of the outbreak.

## Background

An H5 HPAIV outbreak that began in December 2014 and lasted 6 months through June 2015 was the 5th highly pathogenic avian influenza virus (HPAIV) outbreak in the US since the 1920’s and was the most geographically widespread. Direct and indirect costs to the US economy were estimated to be near $3.3 billion USD [1]. The hemagglutinin (HA) gene was determined to belong to clade 2.3.4.4 of the goose/Guangdong/1996 (GS/GD/96) H5 lineage of HPAIV and was subsequently named intercontinental group A (IcA) H5 [2, 3]. Wild waterfowl are thought to have carried viruses of this Eurasian lineage from Asia into North America during migration over the Bearing sea route [2, 4–6]. The GS/GD/96 lineage has circulated in wild and domestic birds throughout Asia since 1996 with occasional incursions into Europe and Africa. In December 2014, the IcA H5 HPAIVs were first discovered in poultry in Canada, and subsequently in the US a few weeks later [4, 7].

In North America four variants of the IcA H5 viruses were identified; an H5N8 with a genome that was completely Eurasian in lineage (≥98% identity among all gene segments with H5N8 virus isolates from South Korea); and three reassortants with a mixture of North American wild bird lineage genes, an H5N2, an H5N1 and an H5N8 [4, 8, 9]. The H5N2 was the variant that caused the most cases in commercial poultry in the US [10]. Turkey farms had the highest number of infected premises (153 of 211) during this outbreak, but table-egg layer chickens were the poultry type with the highest numbers of birds affected [10]. The goal of these studies was to characterize the pathobiology of the US index H5N2 HPAIV isolate collected from a wild Northern Pintail in December 2014 and to compare that with two poultry isolates from later in the outbreak (April 2015) in commercial broad breasted white turkeys.

## Methods

### Turkeys

Broad-breasted white turkeys were obtained from a commercial turkey producer and were reared in a commercial production environment from hatch until they were delivered to the Southeast Poultry Research Laboratory-USDA-ARS (SEPRL) at 4 weeks of age. Each bird was individually tagged for identification. The turkeys were housed and cared for in accordance with procedures approved by the SEPRL Institutional Animal Care and Use Committee. Serum was collected from turkeys immediately prior to challenge to confirm the absence of antibody to type A influenza by commercial ELISA (MultiS Screen, IDEXX Inc. Westbrook, ME).

### Viruses

Three H5N2 HPAIV isolates were selected for evaluation: the US index H5N2 HPAIV isolate from December 2014, A/Northern Pintail/Washington/40964/2014 (NOPI/40964); and two isolates from commercial poultry operations collected in April 2015, A/turkey/MN/12582/2015 (TK/12582) and A/chicken/IA/13388/2015 (CK/13388). Viruses were provided by the National Veterinary Services Laboratories-USDA-APHIS (Courtesy of Dr. Mia Torchetti). Each isolate was passaged twice in embryonating chickens eggs (ECE) and titrated using standard procedures [11]. Inocula were diluted to the appropriate dose in brain heart infusion (BHI) broth.

### Pathogenesis, infectious dose and transmission

To evaluate the infectious dose of each isolate and transmission to non-inoculated hatch-mates housed in the same isolator (contact exposed turkeys), three doses of virus: 10^2^, 10^4^ and 10^6^ 50% egg infectious doses (EID_50_) per bird, were administered to groups of five turkeys as reported in Bertran et al. [12]. Virus was administered using a simulated respiratory route, the intrachoanal route, in 0.1 ml per bird. The contact exposed turkeys (*n* = 3) were added to each dose group 24 h post challenge (PC). Sixteen additional turkeys at the 10^6^ EID_50_ per bird dose group were included to characterize the pathogenesis of the virus. Clinical signs and mortality were recorded a minimum of daily and birds that were euthanized due to severe illness were counted as dying the following day for mean death time (MDT) calculations. Turkeys were considered infected if virus was detected in oro-pharyngeal (OP) or cloacal (CL) swabs at any time point and/or if the bird seroconverted by the end of the experiment (14 days PC).

The later experiments with TK/12582 and CK/13388 had a modified swab collection schedule from what was done with NOPI/40964 because additional sample collection times were added to more precisely establish when the turkeys started to shed detectable levels of virus. For all three isolates, OP and CL swabs were collected from the directly inoculated turkeys at 2, 4, 7, 10 and 14 days PC, and OP and CL swabs were collected from contact exposure turkeys at 1, 3, 6, 9 and 13 days PC. Additional OP and CL samples collected from groups inoculated with TK/12582 and CK/13388 were at 12, 24 and 36 h PC. Virus titers in swabs were evaluated by quantitative real-time RT-PCR. Sera were collected from all surviving turkeys 14 days PC. Sera were tested for antibody by homologous hemagglutination inhibition (HI) assay.

Two to six birds at the 10^6^ EID_50_ per bird dose group exposed to each virus were necropsied for tissue collection when clinical signs were apparent. A full set of tissue samples (lungs, bursa, kidneys, adrenal gland, thymus, bursa, brain, liver, heart, proventriculus, pancreas, intestine, spleen, trachea, Harderian gland, beak, and thigh muscle) were collected for microscopic evaluation and immunohistochemistry (IHC). Tissues were fixed in 10% neutral buffered formalin, sectioned, paraffin embedded, and stained with hematoxylin-and-eosin. Serial sections were stained by IHC methods [13] to visualize influenza antigen in individual tissues. An identical tissue set was collected from two non-inoculated hatch-mates to serve as negative controls.

Sera were collected from all surviving turkeys 14 days PC to evaluate infection status by antibody detection with homologous HI assay.

The infectious dose was calculated by the Reed-Muench method [14], using the criteria that turkeys were considered infected if they had clinical signs, died, shed virus or were positive for antibody 14 days PC.

### Quantitative real-time RT-PCR

Quantitative real-time RT-PCR targeting the influenza M gene including the RNA extraction, was conducted as previously described [15]. The standard curve was run in duplicate using RNA from the same virus stock used to prepare the inocula. Virus quantity was reported as equivalents to infectious titer.

### Hemagglutination inhibition assay

The HI assay was conducted using standard procedures [11]. Homologous isolates were used as antigens and were inactivated with 0.1% beta-propiolactone. A titer of 1:16 (2^4^) or above was considered positive.

### Statistics

Virus titers shed by time point for the same swab type (OP or CL) were tested among the viruses with Kruskal-Wallis H test (Prism 7, GraphPad Software, La Jolla, CA). A *p* value of ≤0.05 was considered significant.

## Results

### Infectious dose and transmission

Infection status was determined based on virus shed, clinical disease, mortality and seroconversion. Turkeys which survived to 14 days were not infected as indicated by lack of virus shed and seroconversion. At each dose and virus combination either 100% of the turkeys were infected or none were infected (Table 1). The 50% turkey infectious dose (TID_50_) and 50% turkey lethal doses (TLD_50_) were the same for each isolate and were approximately 10^5^EID_50_/bird for NOPI/40964 and TK/12582 but was two log_10_ lower for CK/13388 which was 10^3^ EID_50_/bird (Table 1).

**Table 1:** Mortality, mean death time, 50% infectious dose, 50% lethal dose and number of birds shedding for three H5N2 highly pathogenic avian influenza viruses in 4-week old directly inoculated and contact exposed broad-breasted white turkeys

Isolate	Dose (EID_50_/bird)	Mortality		Mean death time (days)		Number of turkeys shedding		Seroconversion		50% Turkey infectious dose^e^
		Inoculated	Contact exposed	Inoculated	Contact exposed	Inoculated	Contact exposed	Inoculated	Contact exposed	
A/northern pintail/WA/40964/2014	10^2^	0/5^a^	0/3^a^	NA^b^	NA	0/5^d^	0/3^d^	0/5^i^	0/3^i^	10^5^ EID_50_^f^
	10^4^	0/5	0/3	NA	NA	0/5	0/3	0/5	0/3	
	10^6^	5/5	3/3	5.3 (4–7)^gh^	7.6 (7–8)^cg^	5/5	3/3	NA	NA	
	10^6^	16/16	NA		NA	16/16	NA	NA	NA	
A/turkey/MN/12582/2015	10^2^	0/5	0/3	NA	NA	0/5	0/3	0/5	0/3	10^5^ EID_50_
	10^4^	0/5	0/3	NA	NA	0/5	0/3	0/5	0/3	
	10^6^	5/5	3/3	5.9 (3–10)	8.0 (6–10)	5/5	3/3	NA	NA	
	10^6^	16/16	NA		NA	16/16	NA	NA	NA	
A/chicken/IA/13388/2015	10^2^	0/5	0/3	NA	NA	0/5	0/3	0/5	0/3	10^3^ EID_50_
	10^4^	5/5	3/3	5.4 (1–7)	9.0 (6–10)	3/5	3/3	NA	NA	
	10^6^	5/5	3/3	5.6 (3–13)	7.6 (2–13)	5/5	3/3	NA	NA	
	10^6^	16/16	NA		NA	16/16	NA	NA	NA	

^a^Number dead/total

^b^ *NA* not applicable. Either no mortality or no birds survived for antibody testing

^c^Calculated from the day of placement with the inoculated turkeys

^d^Number shedding/total

^e^50% infectious dose was equal to the 50% lethal dose for all isolates

^f^EID_50_ = 50% egg infectious dose

^g^Mean death time days (range of mortality in days). For inoculated birds includes data from all 21 turkeys exposed to 10^6^ EID_50_/bird

^h^Mean death times are combined for directly exposed turkeys in the same dose group for each isolate

^i^Number seroconverted/total

Infection rates in the contact exposure groups were the same as the inoculated groups to which they were exposed; 100% of the contact exposure turkeys, with all three isolates, at 10^6^EID_50_/bird dose group were infected. The only lower dose group where the contact turkeys were infected was the 10^4^EID_50_/bird dose group exposed to CK/13388, where 100% (3 of 3) were infected. All the infected contact exposed birds presented with clinical signs similar to the inoculated turkeys and died.

### Virus shed

No turkeys exposed to 10^2^EID_50_/bird shed detectable levels of virus at any time. Only one group exposed to 10^4^EID_50_/bird shed detectable levels of virus: the CK/13388 exposed group (Table 1). In this group, virus was only detected in OP swabs from 3 of 5 turkeys and in CL swabs from 2 of 5 turkeys, although the two turkeys from which virus was not detected both presented with clinical signs consistent with HPAIV and died (one at 24 h PC and the other at 5 days PC).

**Fig. 1:**
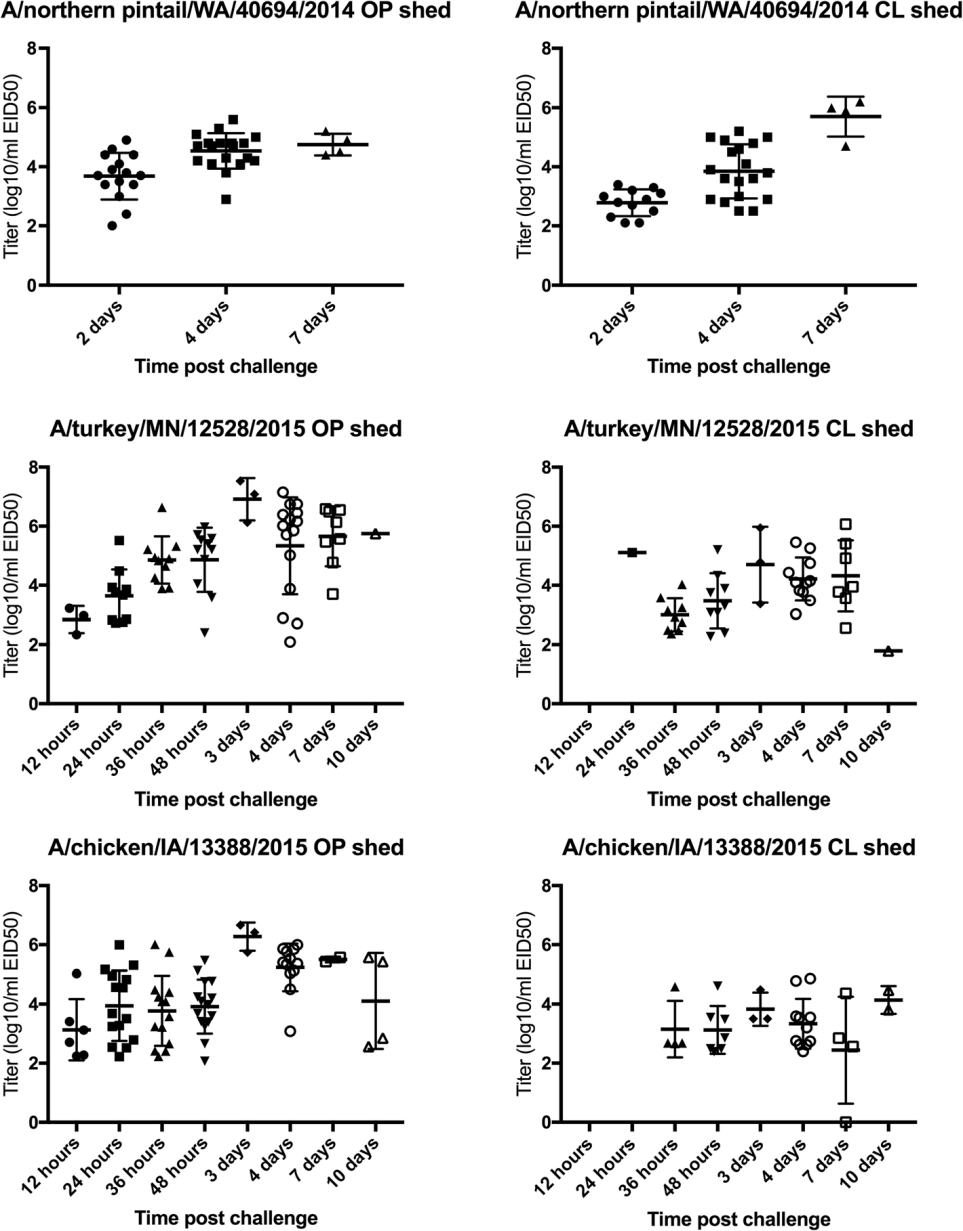
Oropharyngeal (OP) and cloacal (CL) shed determined by real-time RT-PCR for each virus by time post challenge with 10^6^ 50% egg infectious doses (EID_50_) per bird. Data are absent from the A/northen pintail/WA/40964/2014 isolates at 12, 24, 36 h and 3 days because the samples were not collected, at 10 and 14 days because no turkeys survived to these time points. *Thick bars* indicate the mean of the group and *error bars* represent one standard deviation

Oro-pharyngeal and CL shed titers for groups exposed to 10^6^EID_50_/bird are shown in Fig. 1. Oro-pharyngeal shedding was detectable at all times and peaked 3–4 days PC for all three isolates with titers exceeding 10^6^EID_50_/ml from birds exposed to TK/12528 and CK/13388. Although titers from birds exposed to NOPI/40964 were about one log_10_ lower the difference was not significant. Cloacal shedding also peaked at 3 days PC from birds exposed to TK/12528 and CK/133388, but was later for NOPI/40964 exposed birds where the highest titers were at 7 days PC. Turkeys in the 10^6^EID_50_/bird dose group that survived to 10 days PC were still shedding virus. There were no differences in the numbers of turkeys shedding among the isolates for the 10^6^EID_50_/bird dose group (all were 100%).

Shed titers were compared among all three isolates for all turkeys exposed to 10^6^EID_50_/bird by swab type at 2 and 4 days PC and between TK/12582 and CK/13388 at 12, 24, 36 h and 3DPC (at time points where five or fewer birds were shedding detectable levels of virus statistical analysis was not performed). At 36 h PC the OP titers from TK/12528 were significantly lower than CK/13388. Titers of shed among the viruses were only significantly different at two other times; CK/13388 OP shed titers were significantly lower than either NOPI/40964 or TK/12582 at 2 days PC, and CK/13388 titers were again significantly lower than either other virus with CL at 4 days PC.

Each group of contact exposed turkeys shed virus similarly to the inoculated groups with which they were housed. In the groups housed with the 10^6^EID_50_/bird dose group all (3 of 3) contact exposure turkeys shed virus. In the 10^4^EID_50_/bird dose group for CK/13388, which was the only middle or lower dose group to be infected; all three turkeys shed virus (data not shown).

### Pathogenesis

The pathogenesis for all three isolates was similar. Mean death times for the inoculated turkeys were between 5.3 and 5.9 days and were between 7.6 and 9 days post placement for the contact exposed turkeys (Table 1). Clinical disease was not apparent until 24 h or less prior to death (turkeys that could not reach food or water were euthanized). With all three isolates, an average of 50% of the turkeys died without exhibiting any clinical signs within 12 h prior to death (i.e. they appeared normal at the late afternoon/evening observation period and were dead at the morning observation period).

**Fig. 2:**
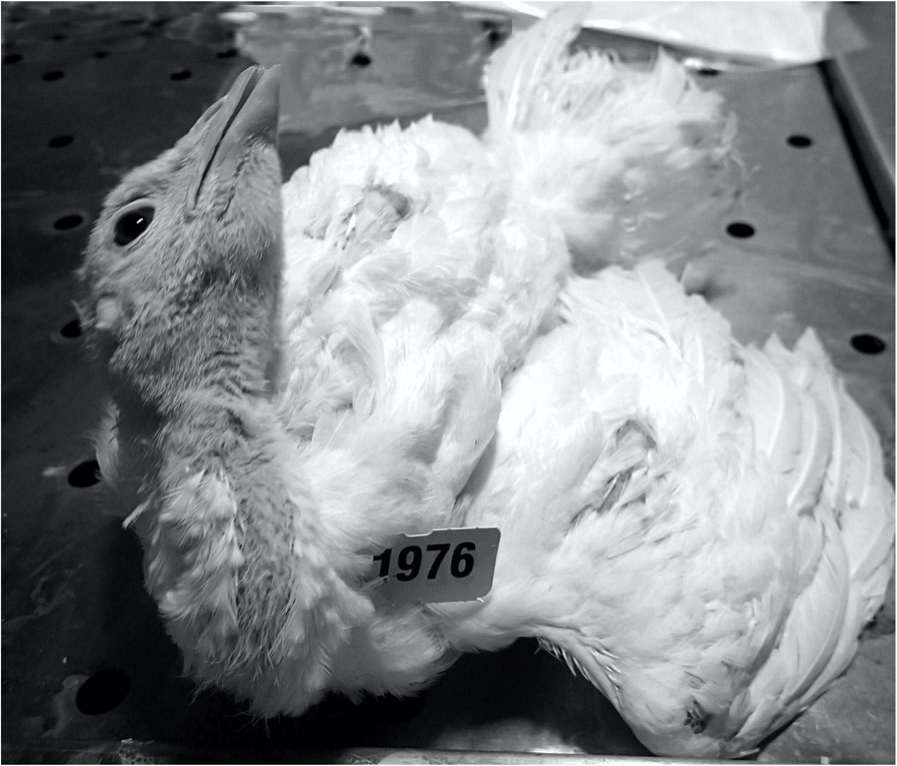
Four week-old broad-breasted white turkey with torticollis after exposure to A/northern pintail/WA/40964/2014 H5N2 highly pathogenic avian influenza virus

The only clinical signs observed were neurological signs and lethargy. Lethargic birds tended to rest with ruffled body feathers. The severity of neurological signs and lethargy was similar among all three isolates. Neurological signs consisting of torticollis (Fig. 2), tremors or ataxia were observed in 28 and 23% of the turkeys infected with NOPI/40964 and TK/12582, and 50% of the turkeys infected with CK/13388.

Turkeys were selected for necropsy because they were presenting with clinical signs (neurological and/or lethargy). Tissues were collected from six turkeys exposed to NOPI/40964 (4 DPC) and from two turkeys exposed to TK/12582 and two turkeys exposed to CK/13388 (3DPC). Gross lesions were typically absent, however petechial hemorrhages were observed in the skeletal muscle of two turkeys: one infected with TK/12582 and CK/13388 each. The CK/13388 infected turkey with the muscle hemorrhage also had swollen kidneys. A third turkey, infected with TK/12582, also had swollen kidneys. Other lesions were non-specific and were consistent with anorexia and lethargy (i.e. approximately 10% of the turkeys had empty alimentary tracts). In addition, any turkeys dying or that were euthanized were necropsied, but no tissues were taken. Gross lesions were all similar to those observed in the turkeys from which tissues were collected.

**Fig. 3:**
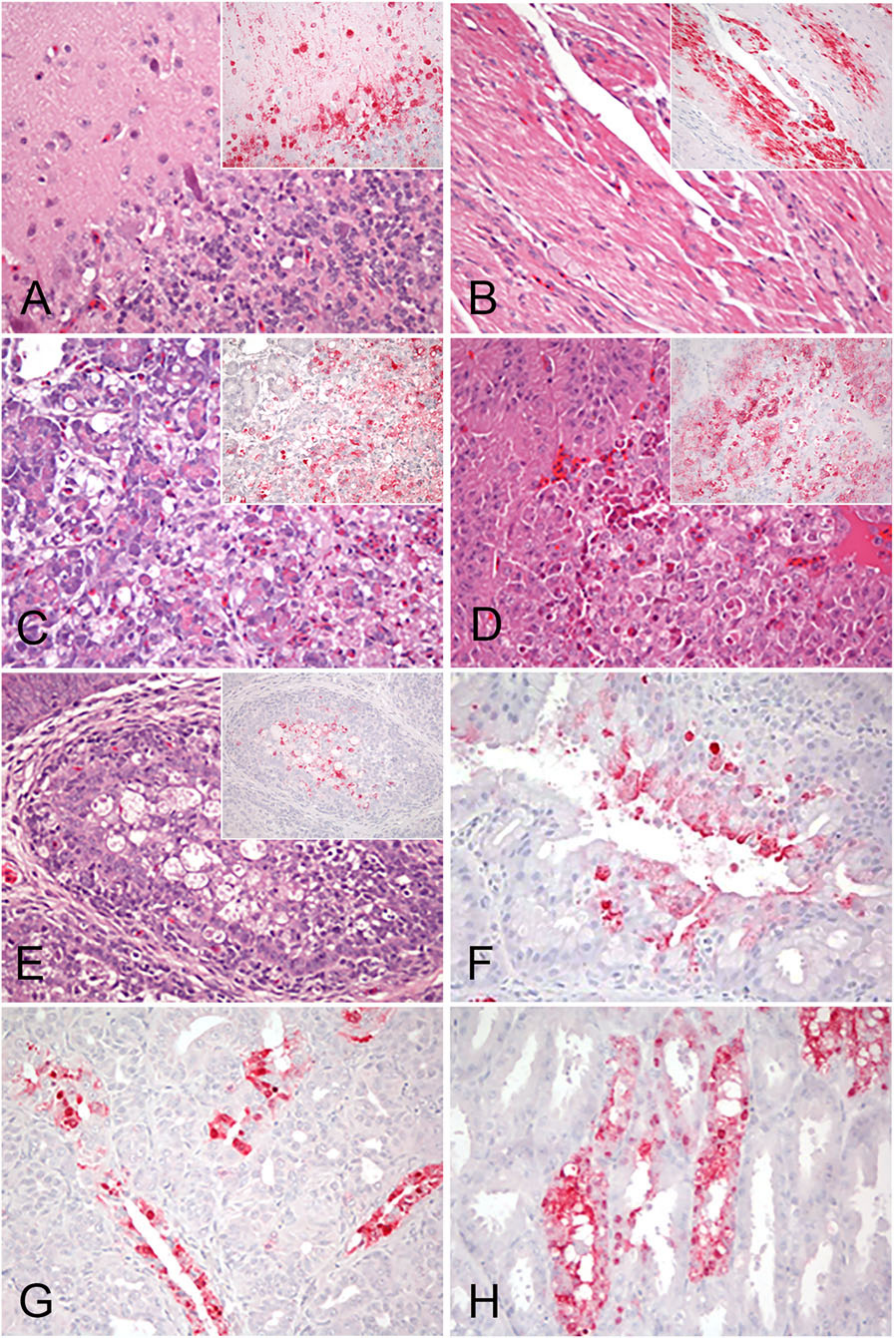
Histological lesions and immunohistochemical detection of viral antigen in 4-week-old turkeys intranasally inoculated with H5N2 HPAI viruses. Tissues collected at 4 days post-inoculation. Virus antigen is stained in *red*. Magnifications 40×. **a** Cerebellum. Vacuolation of the molecular and granular layers of the cerebellum with necrosis of the Purkinge neurons. *Inset*: viral antigen in neurons and glial cells. **b** Heart. Focal hyalinization and fragmentation of cardiac myocytes. *Inset*: viral antigen in cardiac myocytes. **c** Pancreas. Diffuse pancreatic necrosis. *Inset*: viral staining in acinar epithelium. **d** Adrenal gland. Confluent necrosis of corticotrophic and chromaffin cells. *Inset*: viral antigen in adrenal corticotrophic and cromaffin cells. **e** Bursa de Fabricious. Lymphoid depletion with apoptosis to necrosis in remaining lymphocytes. *Inset*: viral antigen in phagocytic c and necrotic cells. **f** Harderian gland. Viral antigen present in epithelial cells and infiltrating phagocytes. **g** Nasal gland. Viral antigen present in glandular epithelial cells and infiltrating phagocytes. **h** Proventriculus. Viral antigen present in glandular epithelial cells

Tissues were examined for microscopic lesions and viral antigen staining (Table 2 and Fig. 3). Microscopic lesions were similar among the eight birds examined, with only minor variations in severity. The most severe lesions were found in the nasal cavity, brain, heart, adrenal gland and pancreas. Moderate to severe rhinitis and sinusitis with multifocal necrosis of the nasal epithelium was common in all birds. Mild tracheitis and bronchitis, and mild to moderate interstitial pneumonia were also present. Mild to severe, randomly disseminated foci of neuronal and glial cell necrosis were observed in the cerebrum and cerebellum of all turkeys examined (Fig. 3a). Microgliosis, edema and mononuclear perivascular cuffs were also commonly observed in malacic areas of the brain. In the heart, mild to moderate multifocal myocyte necrosis was commonly observed (Fig. 3b). Moderate to severe multifocal necrosis of the pancreatic acinar epithelium was present in all birds (Fig. 2c). In the adrenal gland, mild to moderate multifocal to confluent areas of vacuolar degeneration to necrosis of corticotropic cells, and less commonly, chromaffin cells was observed in all but one turkey (Fig. 2d). Thymus, bursa and mucosa-associated lymphoid tissue had moderate to severe lymphoid depletion with apoptosis to necrosis in remaining lymphocytes (Fig. 2e). Mild to moderate depletion of the white pulp with multifocal lymphocytic necrosis was observed in the spleen. Mild to moderate necrosis of the epithelia of the Harderian glands and nasal glands was observed in most turkeys. Infrequently, the kidneys presented mild focal tubular necrosis, and the liver mild multifocal fibrinoid necrosis. Epithelial cell necrosis of the proventricular gland was observed in three turkeys. No lesions were observed in the intestine, eyelid, snood, or skeletal muscle.

**Table 2:** Distribution of avian influenza virus antigen visualized by immunohistochemical staining in tissues by isolate

Bird ID	Detection of avian influenza virus antigen in tissues															
	Nasal epithelium	Nasal glands	Trachea	Lung	Heart	Brain	Liver	Kidney	Adrenal gland	Spleen	Intestine	Pancreas	Harderian gland	Thymus	Bursa	Proventric
A/Northern Pintail/WA/40964/2014																
1043	+	+	−	+	++	+++	+	+	+++	+	+	+++	−	−	+	−
1045	+	++	−	+	+	+++	−	−	+++	−	+	+++	++	++	+	−
1047	+	+	−	+	+	+++	+	+	+++	+	+	+++	+	+	++	+
1052	+	++	−	+	−	+++	+	++	++	−	−	+		+	+	−
1053	+	++	−	−	++	+++	+	+	++	−	++	+++	+	+	++	−
1056	++	++	+	++	++	+++	+	++	+++	−	+++	++	+	++	++	+
A/turkey/MN/12528/2015																
1093	++	+++	−	+	++	+++	−	+	−	−	−	++	+	+	+	−
1097	+	++	+	+	+++	+++	++	+	++	+	−	++	+++	+	+	+++
A/chicken/IA/13388/2015																
1040	+	+	−	+	+++	+++	+	+	+++	+	−	+++	+	−	+	−
1050	+	++	−	+	++	+++	−	+	++	−	−	+++	−	+	++	−

Turkeys were selected for examination because they were presenting with clinical illness at 4 days post challenge (DPC) (A/Northern Pintail/WA/40964/2014) or 3DPC (A/turkey/MN/12528/2015 and A/chicken/IA/13388/2015)

− = no positive cells; + = single positive cells; ++ = scattered groups of positive cells; +++ = widespread staining

Proventric. = proventriculus

Immunohistochemical staining was used to visualize virus distribution in tissues. All three viruses demonstrated similar tissue tropism where the highest levels of viral staining relative to other tissues were observed in the brain, heart, pancreas, and adrenal glands (Table 2). Staining for virus antigen was present in areas of necrosis in many tissues including brain, pancreas, adrenal gland lymphoid tissues, liver, and spleen. Virus antigen was found in parenchymal cells of organs including microglial cells and neurons, cardiac myocytes, pancreatic acinar cells, adrenal corticotropic and chromaffin cells (Fig. 3a-d), hepatocytes, and kidney tubular epithelial cells. Viral staining was common in resident and infiltrating phagocytes of the thymus, bursa and spleen (Fig. 3e). Viral antigen was also present in epithelial cells and macrophage in the nasal turbinates, trachea, Harderian gland (Fig. 3f), nasal glands (Fig. 3g); and proventricular gland cells (Fig. 3h). No viral antigen was observed in vascular endothelial cells.

## Discussion

The pathogenesis of three H5N2 HPAIV isolates from the US 2014–2015 outbreak were characterized in commercial broad breasted white turkeys. These H5N2 isolates, which were first isolated in North America in Canada, represent novel AIV reassortants with genes from Eurasian viruses: a GS/GD/96 derived HA, Eurasian PB2, PA, M and NS genes, and North American wild bid virus lineage PB1, NP, NA genes [7]. The US index isolate, NOPI/40964, was selected to represent the earliest introductions detected in the US and the H5N2 HPAIV variants which are mostly likely to be adapted to wild birds. Two later isolates from the Midwest, TK/12582 and CK/13388, were selected in real-time during the outbreak to represent isolates that had likely been passaged in gallinaceous poultry based on a relatively severe clinical presentation in the field and initial epidemiological information. Based on subsequent sequence analysis and field epidemiology, the TK/12582 isolate was probably an introduction either directly from waterfowl or soon thereafter and may not have been passaged extensively in gallinaceous poultry (unpublished data). In contrast, based on the same analysis, CK/13388 may have circulated in poultry longer. This would corroborate the infectious dose data where the TK/12582 and NOPI/40964 isolates each required a TID_50_ 2 log_10_ higher dose than the CK/13388.

In fact, the TID_50_ and TLD_50_ were the only differences observed among the three isolates. All three primarily presented with neurological signs and severe lethargy during the 24 h prior to death, and virus distribution among the tissues was similar. Microscopic lesions produced by all three isolates were typical for HPAIV in turkeys and other gallinaceous birds. Importantly, a high level of virus was observed in the brain by IHC, which could account for injury to the brain resulting in neurological signs.

Although the clinical presentations among all three isolates were similar, the pathogenesis of these isolates had some unusual characteristics for HPAIV. First, the MDT’s, which were between 5.3 and 5.9 days, and the pre-clinical period were atypically long and mortality was distributed over a 10 day period with some turkeys dying as late as 13 days PC. By contrast, most HPAIV produce MDTs ranging between 2 and 4 days and death has been associated with replication of virus in blood vessel endothelial cells with vascular thrombosis or embolism and multi-organ failure [16, 17]. However, in this study, neural pathogenesis has been associated with direct replication of virus in neurons and neuropil support cells without vascular endothelial cells involvement, and accompanied by replication in other critical organ parenchymal cells leading to multi-organ failure, as has been shown with some previous HPAIV infections [17]. The even longer MDTs with the contact exposed turkeys are most likely because it took a day or two for the turkeys to become infected. Also, since the environment is artificial (e.g. grate floor isolators limiting coprophagy, high rate of airflow, etc.) the results with the contacts cannot be extrapolated directly to the field, and only provide a relative measure of transmissibility among isolates tested in similar ways.

Secondly, each of these viruses was shed at relatively high titers by the cloacal route. Highly pathogenic AIV is more often shed at the highest titers by the oral/respiratory route in gallinaceous birds [16]. It is possible that the late onset of morbidity and mortality, and high virus titers in manure contributed to the rapid spread of the virus in the Midwest, especially in light of the relatively high TID_50_. Essentially the turkeys are infected and are shedding substantial amounts of virus in their manure and orally as early as 24–36 post infection for several days, but don’t appear sick.

One aspect of the field situation that was not replicated in the laboratory was the age of the turkeys. During the outbreak 85% of the infected turkey flocks were at least 9 weeks of age (57% of affected turkeys were 12 or greater weeks of age) [10]. Unfortunately, facilities which can work with HPAIV often cannot accommodate broad-breasted white turkeys in isolation cabinets over about 7 weeks of age because of their size, so it was not possible to determine if turkey age affected susceptibility for biological reasons or whether older birds have a higher chance of exposure because they have been around for a longer period of time (or a combination of both) as animal facilities with the appropriate biosafety level for floor pen studies were not available.

Relatively little data has been reported regarding experimental infection of turkeys with HPAIV and none with isolates from this lineage (clade 2.3.4.4 H5 isolates). Experimental pathogenesis studies in birds have characterized H5N8 HPAIV isolates in chickens and ducks [18–23] and the pathogenesis of NOPI/40964 H5N2 HPAIV in chickens has been reported [12]. The 50% infectious dose in chickens was similar at 10^5.7^ EID_50_ (versus 10^5.0^ EID_50_ in turkeys). Notably there were differences between the pathogenesis of NOPI/40964 in chickens and turkeys: the MDT was 3 days in chickens versus 5.3 days in turkeys and the clinical presentation was also different. Neurological signs were more common in turkeys (only 1 of 10 chickens presented with neurological signs) and there were lesions unique to chickens: 1) petechial hemorrhages and cyanotic combs and wattles (turkey snoods appeared normal); and 2) infra-orbital swelling [12]. Differences in lesions between chickens and turkeys were also observed with H5N2 HPAI in the field in Canada [7] and with a 1997 H5N1 HPAIV GS/GD/96 lineage virus [24]. Similar clinical presentation (i.e. neurological signs) and gross lesions in turkeys in the field were observed in the UK with a GS/GD/96 lineage H5N1 HPAIV [25] and with the unrelated H5N8 HPAIV in Ireland in 1983 [26]. Based on limited information from field reports, lack of respiratory signs is not uncommon in turkeys with HPAIV [7, 25, 26], although respiratory signs were reported with an H5N2 HPAIV in Italy in 1997 [27]. However, in field cases the involvement of secondary bacterial infections cannot be ruled out.

## Conclusions

High mortality is the most consistent sign of HPAIV infection in turkeys as there are some biological variations in the pathogenesis of the virus. In the case of these three isolates, the long pre-clinical period, late MDT and high titers of cloacal shed are unusual characteristics and may have contributed to spread despite the initially high exposure dose to produce infection; i.e. high TID_50_. Also, neurological signs were the most common clinical sign after severe lethargy observed in the turkeys, which contrasts chickens, where hemorrhagic lesions are reported more frequently. This and a shorter reported MDT in chickens highlights the difference in pathogenesis of HPAIV between chickens and turkeys. Finally, there are many other factors involved in the spread of HPAIV in poultry; attention to biosecurity should be a priority regardless of the characteristics of the lineages involved.
